# Grayscale image recording on Ge_2_Sb_2_Te_5_ thin films through laser-induced structural evolution

**DOI:** 10.1038/srep42712

**Published:** 2017-02-14

**Authors:** Tao Wei, Jingsong Wei, Kui Zhang, Hongxia Zhao, Long Zhang

**Affiliations:** 1 Key Laboratory of Materials for High Power Laser, Shanghai Institute of Optics and Fine Mechanics, Chinese Academy of Sciences, Shanghai 201800, PR China; 2 University of Chinese Academy of Sciences, Beijing 100049, People’s Republic of China; 3 Laboratory for High Density Optical Storage, Shanghai Institute of Optics and Fine Mechanics, Chinese Academy of Sciences, Shanghai 201800, PR China

## Abstract

Chalcogenide Ge_2_Sb_2_Te_5_ thin films have been widely exploited as binary bit recording materials in optical and non-volatile electronic information storage, where the crystalline and amorphous states are marked as the information bits “0” and “1”, respectively. In this work, we demonstrate the use of Ge_2_Sb_2_Te_5_ thin films as multi-level grayscale image recording materials. High-resolution grayscale images are recorded on Ge_2_Sb_2_Te_5_ thin films through taking advantage of laser-induced structural evolution characteristic. Experimental results indicate that the change of laser energy results in the structural evolution of Ge_2_Sb_2_Te_5_ thin films. The structural evolution induces the difference of electronic polarizability and reflectivity, and high-resolution grayscale images are recorded on Ge_2_Sb_2_Te_5_ thin films through direct laser writing method, accordingly.

The binary bit encoding is extensively exploited in traditional optical data storage^1^. However, the binary bit encoding actually has the disadvantages of low storage density and complex encoding transfer for read-out. For example, when an image is stored by binary encoding method, the storage capacity of several gigabits is generally required. In contrast, if the image can be recorded through multi-level bit data encoding method, the capacity may be improved greatly compared with traditional binary encoding method^2^,^3^. The multi-level bit encoding can be realized through direct grayscale recording^4^,^5^. Moreover, the grayscale image recording has the advantage of facile writing and readout without the transcoding process. Chalcogenide Ge_2_Sb_2_Te_5_ thin films, possessing good thermal stability and large number of achievable rewriting cycles^6^,^7^,^8^,^9^, have been successfully applied in binary bit encoding data storage by taking advantage of the optical reflectivity contrast between the crystalline and amorphous states induced by laser pulse radiation^6^,^10^,^11^. Ge_2_Sb_2_Te_5_ thin films have also been used for fluorescence phase-change multilevel recording medium^12^. Recently, Wang, *et al*. demonstrated high-density multi-level crystallization of a Ge_2_Sb_2_Te_5_ thin film by adjusting focused femtosecond laser pulse number^13^. Moreover, grayscale images were written into the storage medium. However, details of structure revolution are less reported for grayscale image recording.

Ge_2_Sb_2_Te_5_ thin film is used as our grayscale recording material, which has the crystallization temperature of about 160 °C^13^. Low laser energy is required for grayscale recording. Moreover, large change of optical reflectivity of Ge_2_Sb_2_Te_5_ thin film is also beneficial for the realization of colorful grayscale^6^. Our prior work reported the use of Sb_2_Te_3_ thin film as grayscale recording material^4^. The formation of grayscale image is due to different height and size of bump structure. In this work, it is found that the change of laser energy can result in the evolution of chemical bonds of Ge_2_Sb_2_Te_5_ thin films, which induces the difference of electronic polarizability and then reflectivity change. Thus, multi-level bit encoding features can be easily generated only by tuning laser energy, and one can obtain the grayscale image recording on Ge_2_Sb_2_Te_5_ thin films through taking advantage of the laser-induced structural evolution. Arbitrary images such as tiger, dog and flowers have been successfully recorded on Ge_2_Sb_2_Te_5_ thin films by direct laser writing method.

## Experimental Details

The Ge_2_Sb_2_Te_5_ thin film deposited on a K9 glass substrate with the thickness of 100 nm was prepared by radio frequency magnetron controlling sputtering system (JGP560 type) at room temperature, in which the background pressure was approximately 7.5 × 10^−4^ Pa, the sputtering power was 30 W and the working gas was Ar gas with the pressure of 0.5 Pa.

The grayscale image recording was obtained by direct laser writing system (LASERWRITER LW405B+) with the laser wavelength of 405 nm and the numerical aperture (NA) of 0.65 for focusing lens. The recorded spot diameter was theoretically 760 nm based on the diffraction limit formula D = 1.22 × λ/NA, where D was diameter of spot; λ was laser wavelength and NA was numerical aperture of focusing lens. In practice, the spot size was 1 μm due to the imperfect optical system. The image recording processes are as follows: the grayscale values of each pixel of original pictures are read by computer program. Afterwards, each grayscale value corresponds to a laser power value. Then computer program controls the laser power of each pixel by grayscale values. Thus, arbitrary grayscale patterns are fabricated by continuously adjustable laser power value. In writing process, beam scanning mode was utilized, that is, laser beam moved on the sample surface by raster scanning and performed scanning writing in line by line. X-ray diffraction (XRD) data were recorded via 18KW-D/MAX2500V type equipment produced by Rigaku. The fabricated grayscale patterns were observed by optical microscope (Olympus BX51 microscopy) with white light source illumination. In order to investigate the mechanism of multi-level grayscale image recording, micro-reflection and Raman spectra between laser-irradiated and un-irradiated regions were obtained by reflection spectrum (PG2000-Pro, Idea Optics Company, China) and Renishaw Micro-Raman Spectroscopy System with the excitation wavelength of 532 nm, respectively. X-ray photoelectron spectroscopy (XPS) measurement was performed by an XPS spectrometer (Microlab 310F) produced by VG Scientific and the exciting source is Mg Kα. Optical constant of prepared Ge_2_Sb_2_Te_5_ layers were obtained from the analysis of spectroscopic ellipsometry data measured using an ellipsometer with an automatic rotating analyzer (VASE, J. A. Woollam Co., Inc.). All measurements were carried out at room temperature.

## Results and Discussion

### Multi-level grayscale features

**Figure 1:**
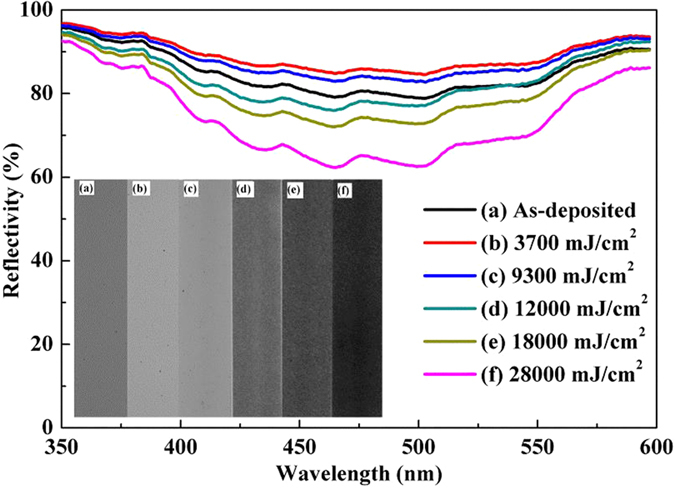
Reflective spectra (inset: multi-level grayscale tones) of Ge_2_Sb_2_Te_5_ thin films at the irradiation of various laser energy densities, (**a**) as-deposited state; (**b**) 3700 mJ/cm^2^; (**c**) 9300 mJ/cm^2^; (**d**) 12000 mJ/cm^2^; (**e**) 18000 mJ/cm^2^; (**f**) 28000 mJ/cm^2^.

For the purpose of achieving grayscale image recording on Ge_2_Sb_2_Te_5_ thin films, multi-level grayscale characteristics are obtained by tuning laser energy density. Moreover, reflective spectra are determined as can be seen in Fig. 1, where Al mirror is firstly used to correct the base reflectivity and its reflectivity is set to 100%. It can be observed from the inset of Fig. 1 that with increasing laser energy from 0 to 3700 mJ/cm^2^, the color of Ge_2_Sb_2_Te_5_ thin film becomes lighter and then it becomes darker when further increasing laser energy from 3700 mJ/cm^2^ to 28000 mJ/cm^2^. From reflective spectra one can see that when the laser energy is 3700 mJ/cm^2^, the reflectivity reaches the maximum and further increase of laser energy density from 3700 mJ/cm^2^ to 28000 mJ/cm^2^ leads to gradual reduction of reflectivity. The reflectivity difference can be as high as 20%, which in turn may generate large optical contrast. Results suggest that multi-level grayscale tones can be determined by finely tuning laser energy and Ge_2_Sb_2_Te_5_ thin film is a promising candidate material for grayscale image recording. It is noted that the grayscale in Fig. 1f becomes black while corresponding lowest reflectivity still reaches to 60%. This may be due to the fact that strong light source illumination in optical microscopy generates strong mirror reflection and high reflectivity. Further study will be performed to increase absolute contrast for the applications of real image recording.

### Laser induced structural evolution characteristic

#### X-ray diffraction analysis

**Figure 2:**
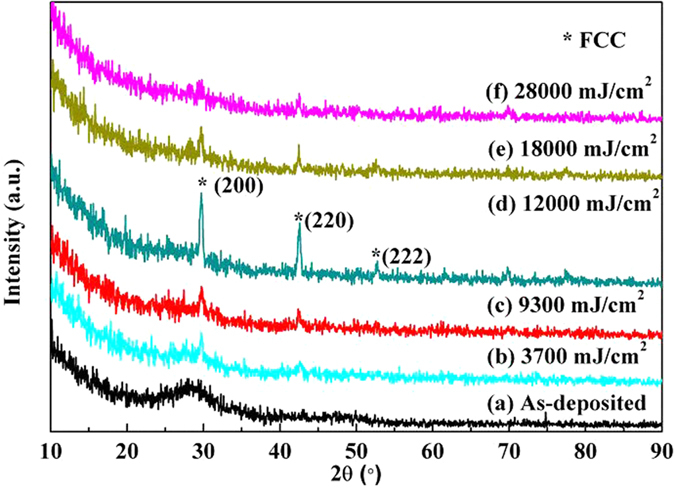
X-ray diffraction patterns of the Ge_2_Sb_2_Te_5_ thin films with various laser energy irradiations, where (**a**) as-deposited state; (**b**) 3700 mJ/cm^2^; (**c**) 9300 mJ/cm^2^; (**d**) 12000 mJ/cm^2^; (**e**) 18000 mJ/cm^2^ and (**f**) 28000 mJ/cm^2^.

Figure 2 shows the X-ray diffraction patterns of Ge_2_Sb_2_Te_5_ thin films. It can be seen that only a small mountain peak centered at 28° is observed in as-deposited Ge_2_Sb_2_Te_5_ thin film, indicating that the prepared thin film is almost amorphous state. After the laser energy irradiation, the Ge_2_Sb_2_Te_5_ thin film with obvious diffraction peaks is determined, which corresponds to a typically metastable face-centered cubic crystal^14^. Moreover, the diffraction peaks become stronger with the increment of laser energy density from 3700 to 12000 mJ/cm^2^. However, when the laser energy further increases to 28000 mJ/cm^2^, the diffraction peaks become weaker gradually. This phenomenon is similar to other reports^15^ and can be explained that the thin film is excessively ablated after the laser irradiation and then transformed back to an amorphous state due to high laser energy density.

#### Raman spectra analysis

**Figure 3:**
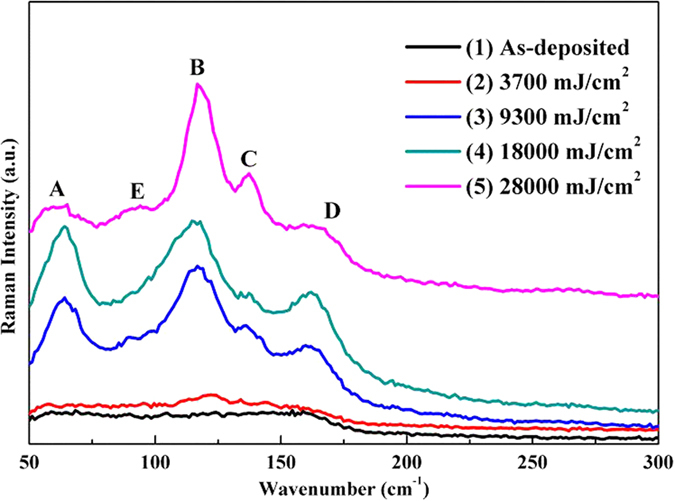
Raman spectra of Ge_2_Sb_2_Te_5_ thin films with the irradiation of various energy densities: (1) as-deposited sample; (2) 3700 mJ/cm^2^; (3) 9300 mJ/cm^2^; (4) 18000 mJ/cm^2^; (5) 28000 mJ/cm^2^.

To further characterize the structure changes, Raman spectra of laser-irradiated Ge_2_Sb_2_Te_5_ thin films are measured as shown in Fig. 3. One can see that the as-deposited thin film shows a broad peak around 150 cm^−1^ which is a characteristic peak for amorphous Ge_2_Sb_2_Te_5_ film and normally ascribed to the stretching vibration of amorphous Te–Te bond^14^,^16^,^17^. After the laser irradiation, the broad peak at 150 cm^−1^ is gradually divided into several peaks marked as ‘A’, ‘B’, ‘C’ and ‘D’. Raman peak ‘A’ is located at 64 cm^−1^, which is very similar to other reports and can be assigned to the vibration of 3-fold coordinated Te^18^,^19^. The peaks ‘B’ at 117 cm^−1^ are very similar to the results in ref. 20, in which the Raman peak is located at 120 cm^−1^ and assigned to A_1_ symmetry of corner-sharing GeTe_4−n_Ge_n_ (n = 0,1, 2, 3) tetrahedral units^14^,^15^,^21^,^22^. The difference may be caused by the vibration of heteropolar bonds in GeTe_4_ or GeTe_3_Ge^15^. The peak ‘C’ at 137 cm^−1^ may be attributed to the segregation of Te crystalline phase for over-irradiation^14^. The peak ‘D’ at 160 cm^−1^ can be ascribed to the A_1g_(2) vibration of crystalline Sb_2_Te_3_^15^.

With increasing laser energy from 3700 to 28000 mJ/cm^2^, the vibrational peaks at 117 cm^−1^ and 137 cm^−1^ become stronger gradually. However, the peak at 160 cm^−1^ becomes weaker and has been dramatically suppressed when the laser energy is up to 28000 mJ/cm^2^. This indicates that the formation of Te crystal is at the expense of crystalline Sb_2_Te_3_, and leads to a large suppression of peak at 160 cm^−1^. Besides, when laser energy is up to 28000 cm^−1^, a new Raman peak ‘E’ at 90 cm^−1^ can be observed, which is due to the E mode of GeTe_4_ tetrahedral^22^.

#### XPS spectra analysis

**Figure 4:**
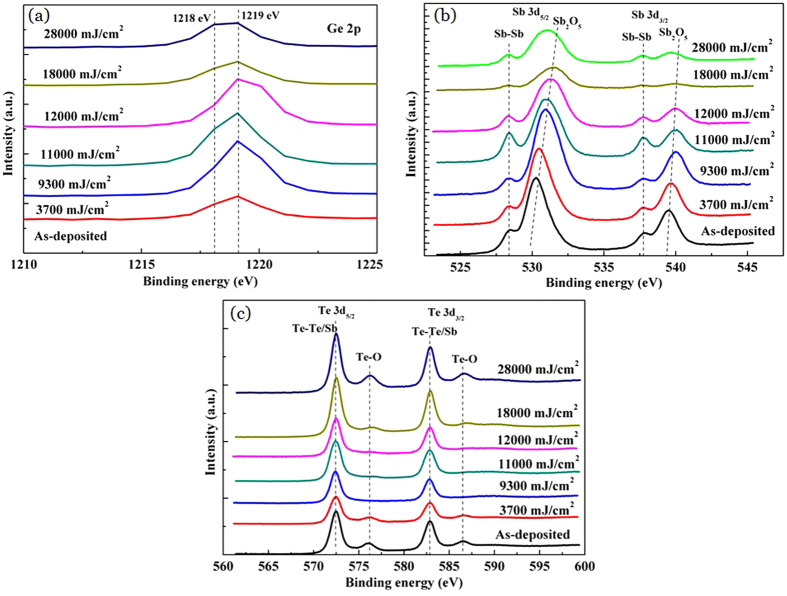
XPS spectra at the irradiation of various laser energy densities; (a) Ge 2p, (b) Sb 3d, (c) Te 3d.

XPS was used for the determination of the binding state of each component as Ge, Sb and Te. Figure 4a displays XPS spectra of Ge 2p_3/2_. One can see a main peak at 1218 eV, which is assigned to Ge-Sb or Ge-Te metallic bonds in as-deposited Ge_2_Sb_2_Te_5_ thin films^23^,^24^. With increasing the irradiation of laser energy, the main peak position moves to 1219 eV, indicating the formation Ge-O bonds^24^. Moreover, the signal intensity of Ge-O bond increases to the maximum when the laser energy is up to 12000 mJ/cm^2^. However, further increment of laser energy leads to weaker Ge-O bond signal and stronger Ge-Sb or Ge-Te bond signals.

Figure 4b shows XPS spectra of Sb 3d at the irradiation of various laser energy densities. For the as-deposited sample, there are four peaks at 528.48 eV, 530.28 eV for Sb 3d_5/2_ and 537.78 eV, 539.48 eV for Sb 3d_3/2_, respectively. Among them, the peaks at 528.48eV and 537.78eV are ascribed to the homopolar (Sb-Sb) bonds while peaks at 530.28 eV and 539.48 eV are due to the Sb-O bonds due to the strong surface oxidation effects^25^,^26^. With increasing laser energy, the signals of Sb-Sb bonds become stronger. When laser energy density is up to 12000 mJ/cm^2^, the intensity of Sb-Sb bond reaches the maximum, which indicates that Sb homopolar bonds play an important role to maintain overall structure stability in crystalline phase. However, further increasing laser energy causes weaker signals of Sb-Sb bonds. In addition, the peak of Sb-O bond moves to higher energy region with the increment of laser energy, which may result in multi-level grayscale on Ge_2_Sb_2_Te_5_ thin films due to obvious structural changes.

The XPS spectra of Te 3d are depicted in Fig. 4c. In general, the peaks located at 572.5~574 eV and 583~584 eV were related to Te metallic bonds^25^. However, in our work, the peaks of metallic bonds are located at 572.48 eV and 582.88 eV, respectively. This may be ascribed to Sb-Te and Te-Te bonds^25^,^26^. When laser energy increases, the Te metallic bonds have no evident changes. Additionally, the binding energies peaks at 576.28 eV and 586.68 eV both correspond to Te-O bonds^26^. It can be seen that the peaks of Te-O bonds gradually become weaker as laser energy increases and then disappear totally at the laser energy of 9300~12000 mJ/cm^2^. Further increment of laser energy leads to the formation of Te-O bonds, again. The disappearance of Te-O bonds can be due to that Te atoms participate in the formation and growth of crystalline Ge_2_Sb_2_Te_5_ thin films.

#### Spectroscopic ellipsometry analysis

**Figure 5:**
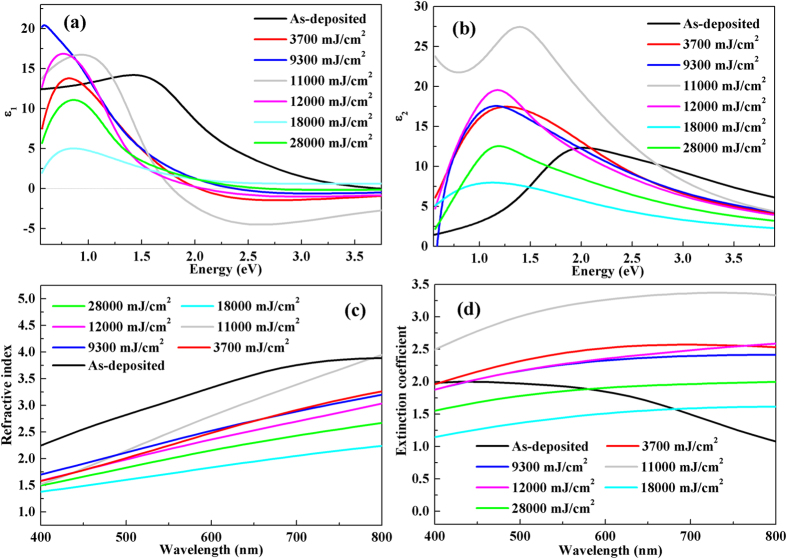
The (a) real (ɛ_1_) and (b) imaginary (ɛ_2_) part of the dielectric functions; (c) refractive index and (d) extinction coefficient in Ge_2_Sb_2_Te_5_ thin films at the irradiation of various laser energy densities.

In order to more intuitively understand the chemical bonding origin of optical contrast between as-deposited state and laser irradiated regions, spectroscopic ellipsometry data are determined and wavelength dependent dielectric functions, refractive index and extinction coefficient are shown in Fig. 5. The detailed data processing can be found elsewhere^21^,^27^. It can be seen from Fig. 5(a and b) that the dielectric functions show a single peak due to bonding–antibonding transitions^10^. Furthermore, ɛ has substantial differences at peak position and peak shape between as-deposited state and laser irradiated regions. Moreover, with changing laser energy, ɛ also has evident changes, which indicates that electronic polarizability and chemical bonds vary with tuning laser energy density^10^,^28^,^29^. In parallel, the refractive index and extinction coefficient change substantially with adjusting laser energy as revealed in Fig. 5(c and d). This in turn brings about the evolution of optical reflectivity. However, the tendency of refractive index and extinction coefficient change is in poor agreement with the trend of optical reflectivity change. It may be explained that the optical reflectivity is determined by the combination of refractive index and extinction coefficient. Therefore, it is proposed that multi-level grayscale image recording may be realized by tuning laser energy.

#### Multi-level grayscale image recording

**Figure 6:**
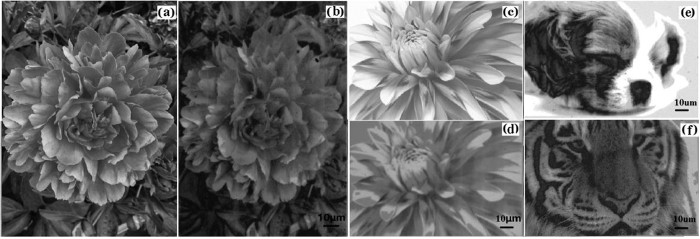
Grayscale patterns written on Ge_2_Sb_2_Te_5_ thin films with the energy density of 93000 mJ/cm^2^, where (a) and (c) are original pictures and (b), (d–f) are recording grayscale images. It is noted that (**a**–**d**) are flowers; (**e**,**f**) are dog and tiger patterns.

According to tuning laser energy, multi-level grayscale image recording can be achieved. The arbitrary multi-level grayscale images are recorded on Ge_2_Sb_2_Te_5_ thin films as shown in Fig. 6. The grayscale levels used in the recorded grayscale images were reached to 256 levels. It is noted that the laser energy density ranges from 0 to 93000 mJ/cm^2^. Figure 6(b and d) show the lifelike flowers, in which the details can be presented clearly and very similar to original pictures as shown in Fig. 6(a and c). In addition, Fig. 6(e and f) present dog and tiger images recorded on the Ge_2_Sb_2_Te_5_ thin film, respectively. Figure 6(e) displays recorded patterns of the sleeping dog. The expression and appearance of the sleeping dog can be clearly recorded on the Ge_2_Sb_2_Te_5_ thin film. Besides, one can see from Fig. 6(f) that the expression and appearance of the lifelike tiger are fully recorded onto Ge_2_Sb_2_Te_5_ thin films by precisely controlling the laser energy density, which can be observed from some fine structures like eyes, furs and whiskers. It is worthwhile mentioning that the regions in Fig. 6(c) appear brightest; however the corresponding regions in Fig. 6(d) appear darker. This is due to that the laser energy density is not optimal. Further optimization of laser energy density will be performed to obtain more lifelike grayscale images. Results demonstrate that Ge_2_Sb_2_Te_5_ thin film is a promising multi-level grayscale image recording material for applications of high-resolution grayscale image storage.

## Conclusions

In summary, we demonstrate that the change of laser energy results in the structural evolution of Ge_2_Sb_2_Te_5_ thin films. The structural evolution induces the difference of electronic polarizability and then reflectivity change. It is found that the reflectivity increases with enhancing crystallization extent of Ge_2_Sb_2_Te_5_ thin film; however, further increasing laser energy leads to the molten state of thin films and lower reflectivity. The combination of amorphous, crystallization and molten state generates colorful grayscale levels. The multi-level bit encoding features can be generated only by tuning laser irradiation energy. The grayscale image recording on Ge_2_Sb_2_Te_5_ thin films can be obtained through taking advantage of the laser-induced structural evolution, accordingly. Arbitrary images have been successfully recorded on Ge_2_Sb_2_Te_5_ thin films, which indicate that Ge_2_Sb_2_Te_5_ thin film is a promising candidate for applications of high-resolution grayscale image storage.

## Additional Information

**How to cite this article**: Wei, T. *et al*. Grayscale image recording on Ge_2_Sb_2_Te_5_ thin films through laser-induced structural evolution. *Sci. Rep.* **7**, 42712; doi: 10.1038/srep42712 (2017).

**Publisher's note:** Springer Nature remains neutral with regard to jurisdictional claims in published maps and institutional affiliations.
